# Investigating the optimum timeline for final cure assessment of treated Visceral Leishmaniasis patients in Bangladesh

**DOI:** 10.1371/journal.pgph.0006002

**Published:** 2026-02-26

**Authors:** Soumik Kha Sagar, Shomik Maruf, Rajashree Chowdhury, Md Masud Ur Rashid, M. M. Aktaruzzaman, Md. Nazmul Islam, Sheikh Daud Adnan, Prakash Ghosh, Md Utba Rashid, Md Rasel Uddin, Rupen Nath, Faria Hossain, Debashis Ghosh, Dinesh Mondal

**Affiliations:** 1 Nutrition Research Division, International Centre for Diarrhoeal Disease Research, Bangladesh (icddr, b), Shaheed Taj Uddin Ahmed Sarani, Mohakhali, Dhaka, Bangladesh; 2 Department of Pharmaceutical Sciences, College of Pharmacy, University of Nebraska Medical Center, Omaha, Nebraska, United States of America; 3 National Heart Foundation and Research Institute, Mirpur, Dhaka, Bangladesh; 4 Communicable Disease Control (CDC), Directorate General of Health Services (DGHS), Mohakhali, Dhaka, Bangladesh; 5 Fachgebiet Empirische Gesundheitsökonomie, Department of Empirical Health Economics, Berlin, Germany; 6 Institute of Animal Hygiene and Veterinary Public Health, University of Leipzig, Leipzig, Germany; 7 Department of Epidemiology and Biostatistics, Arnold School of Public Health, University of South Carolina, United States of America; Medecins sans Frontieres, INDIA

## Abstract

Bangladesh became the first country globally to receive validation for the public health elimination of Visceral Leishmaniasis (VL). However, while VL cases have declined, sequels such as treatment failure and relapse are increasing. The current practice of assessing VL cure at six months post-treatment may delay the identification of sequelae. This study aims to determine the time required for VL patients to achieve complete clinical recovery and parasitological clearance, contributing to sustainable elimination efforts. We enrolled 50 confirmed VL patients from VL-endemic subdistricts of Mymensingh between June 2016 and March 2018. All the enrolled patients were treated with a single dose of Liposomal Amphotericin B (LAmB) (10 mg/kg intravenous infusion). Blood samples were collected upon admission and monthly for six months post-treatment. Polymerase chain reaction (PCR) was performed on these samples. The Kaplan-Meier method was used to estimate the cumulative probability of cure time, with clinical and laboratory parameters measured monthly. The median cure time for the participants was one month. Within three months, 90% of the patients were cured, and all patients fully recovered by six months. A significant difference in cure time was noted between individuals with spleen sizes below and above 8 cm (p = 0.0002). Additionally, nutritional improvements were evident, with increased BMI and a decrease in underweight proportions. Hemoglobin levels showed significant improvement in the early months post-treatment, with complete resolution or significant reduction of splenomegaly observed in all patients by three months, prompting reconsideration of the final cure declaration to three months instead of six months after treatment. Adjusting this cure declaration (while retaining 6, 12, 24, 36, 48, and 60 months of follow-up) will facilitate early detection of non-responsive cases and optimize resource allocation crucial for sustaining VL elimination efforts.

## Introduction

Leishmaniasis is a deadly vector-borne illness caused by over 20 *Leishmania* species, resulting in various disease presentations, extending from localized skin lesions to potentially fatal systemic conditions, such as visceral leishmaniasis (VL) [1,2]. Factors such as poor living conditions, inadequate housing, environmental hygiene, economic disparities, gender inequality, conflict-induced migrations, weakened immune systems, and insufficient nourishment contribute to its prevalence [3]. VL, commonly known as Kala-azar, has been a global public health problem, especially in the Indian subcontinent (ISC) until recently [4]. It is primarily caused by the *Leishmania donovani* complex in Asia and Eastern Africa [5] and follows an anthroponotic cycle for transmission in the ISC [6]. If untreated, the disease is fatal, with outbreak potential. Globally, around 50,000–90,000 new VL cases occur annually, with only 25% to 45% of them reported to the World Health Organization (WHO) [1]. Bangladesh, India, and Nepal, three highly endemic countries, signed a Memorandum of Understanding (MoU) in 2005, intending to eliminate VL by 2015 by launching a kala-azar elimination program (KEP) that extended to 2020 [7]. This initiative brought a decrease in new VL cases in the region, resulting in a 77% reduction in global VL incidence in 2019 [8]. Bangladesh achieved the goal of reducing VL cases to < 1/10000 population at the upazila (sub-district) level in 2017 and was validated as the first country globally to eliminate VL as a public health problem by the WHO [9,10].

The policies and strategies taken up by the KEP contributed substantially to reducing VL prevalence in the ISC [11]. The KEP relies on five main strategies: early case detection for diagnosis and treatment, disease surveillance, vector control, social mobilization and partnership, and operational research [12]. Effective treatment and subsequent follow-ups are pivotal to improved patient outcomes and limiting disease transmission [13]. In the Indian sub-continent, the single-dose AmBisome has been proven to be highly effective for VL treatment. Nevertheless, to evaluate patient outcomes and treatment success, patients are required to attend follow-up visits at 1 and 6 months after completing treatment to ascertain the initial and final cure of VL, respectivel, followed by five more follow-up visits at 12, 24, 36, 48, and 60 months. If the patient shows no improvement within one month or experiences a recurrence of VL symptoms and signs within six months, the patient is considered a Kala-azar treatment failure case (KATF). Recurrence of symptoms and signs of VL after the final cure (6 months post-treatment) is regarded as a relapse case (VLR) [14]. Post Kala-azar Dermal Leishmaniasis (PKDL), on the other hand, is a non-fatal complication of VL that is frequently observed after the successful completion of VL treatment, but PKDL cases among individuals without any history of VL have also been reported in the past [15]. VL relapse, KATF, and PKDL cases are infective to sandflies, maintaining the spread of the infection and posing a threat to the control of VL [16,17].

Despite the success of the National Kala-azar Elimination Programme (NKEP) in constraining active VL transmission, the sequelae of VL are on the rise. In 2019, out of 223 treated cases of leishmaniasis, 33 (14.8%) cases were either KATF or VLR. The number rose to a staggering 25% (24 out of 99) in 2021. To sustain the VL elimination achievement, these patients must be diagnosed and treated at the earliest possible time to stop any possible disease transmission, as they could serve as a potential reservoir. According to the current treatment guideline, the final cure assessment for VL is performed six months after treatment, although this has not been validated yet. One of the major drawbacks of such a long, definitive cure time point has been the loss of follow-up. Additionally, at the post-elimination stage for VL, interruption of infection transmission has been regarded as a priority by WHO, which aims at reducing the VL infection to zero in a defined geographical area with minimal risk of reintroduction. The long gap between initial and final cure assessment timelines might cause delayed identification of KATF cases, eventually delaying treatment commencement and resulting in possible disease transmission. In this study, we strived to determine the time required for a treated VL patient to achieve an optimum level in clinical and laboratory parameters, as well as to get parasitological clearance, thus readjusting the current final cure assessment timeline of VL. Additionally, we attempted to validate the current final VL cure time point of six months. Findings from this study will help the national program to reschedule the current final cure assessment timeline of the treated VL patients, which will eventually lead to a sustainable VL elimination effort.

## Methods

### Ethical statement

Both the original (PR-14093) and current (23049) studies obtained ethical approval from the Institutional Review Board (IRB) of the International Centre for Diarrheal Disease Research, Bangladesh (icddr,b). Informed written consents were obtained from the patients/guardians of each participant under 18 years of age.

### Study sites and participants

This current study is part of an endeavor to evaluate the sensitivity and specificity of Loop-mediated Isothermal Amplification (LAMP) and Urinary Antigen ELISA Assay (UAEA) to diagnose VL [18]. We conducted the study in VL endemic sub-districts of Mymensingh between June 2016 and March 2018. Sample collection and template preparation took place at the Surya Kanta Kala-azar Research Center (SKKRC) in Mymensingh, while laboratory tests were conducted at the icddr,b in Dhaka. In the study, suspected VL patients were referred to the Surya Kanta Kala-azar Research Centre (SKKRC) of the Mymensingh Medical College Hospital following field screening for disease. 80 individuals without a prior history of VL, presenting with a fever lasting more than two weeks, splenomegaly, a positive rK39 rapid detection test (RDT), and a positive qPCR (blood) were classified as VL cases and enrolled in the study. After obtaining informed written consent from the patient/legal guardian, the medical officer collected the socioeconomic and clinical history of the patient. Each VL patient received a single intravenous infusion of LAmB at a dosage of 10 mg/kg. Patients were monitored for their response to treatment and the development of post-treatment complications related to visceral leishmaniasis at six months and 12 months after the initiation of treatment.

To meet the objective of the current study, we approached all 80 VL patients on hospital admission to collect their blood samples each month for up to six months after treatment completion to perform qPCR. Among them, 50 participants consented to participate in the study and provided their samples. Others declined to participate primarily due to the frequency of follow-up visits, concerns about frequent blood sampling, and temporary migration for professional work. The samples were collected and stored. Laboratory tests of the stored samples were done, and data were analyzed between 01 June 2023 and 26 December 2023. The definitive VL cure assessment is done at six months according to the national guideline, based on the following criteria:

- No fever.
- Substantially reduced spleen size or not palpable.
- A feeling of general well-being.

However, this study considered a negative qPCR analysis on whole blood and a completely regressed spleen combined with the other two above criteria as a definitive cure.

### Clinical specimens and laboratory techniques

2 ml of the blood sample was used for qPCR. DNA was extracted from 200µL of heparin-treated whole blood (WB-QIA). The extracted DNA was eluted into 200 μL and 150 μL of elution buffer provided with the kits, following the manufacturer's instructions. The extracted DNA samples were then stored at -80°C. Quantitative PCR (qPCR) was conducted using template DNA extracted by the QIAGEN extraction method from both whole blood and dried blood spots (DBS), following a protocol detailed elsewhere [19], which targeted the conserved REPL repeats of the Leishmania genome. In brief, a 20 µL reaction mix was prepared, comprising 5 µL of template DNA, 10 µL of TaqMan Gene Expression Master Mix (Applied Biosystems), 1 µL of pre-ordered TaqMan primer-probe mix (Applied Biosystems), and PCR-grade water. Amplification was carried out using a Bio-Rad CFX96 iCycler system with the following conditions: 10 min at 95°C, followed by 15 seconds at 95°C, and 1 min at 60°C for 45 cycles. Each run included a standard curve ranging from 10 ng to 1 fg of parasite DNA extracted from in vitro cultured promastigotes (*L. donovani* MHOM/IN/80/DD8), corresponding to 10,000 to 0.1 parasites per reaction. Additionally, a negative control reaction with molecular-grade water was included in each assay. Samples with a cycle threshold (Ct) value exceeding 40 were regarded as negative. All samples were analyzed in duplicate, and in case of an indeterminate result, an additional run was conducted.

Details of the study methods, procedures, and results of the main study have been described by Hossain et al. [18]

### Statistical analysis

A meticulously crafted data entry program was established using Microsoft Access 2019, ensuring a robust foundation for data integrity. Before input into this system, we implemented a rigorous double-checking process, strictly adhering to data management guidelines. The subsequent dataset analysis was conducted utilizing STATA version 17 [20]. Descriptive statistics were generated to unveil the inherent characteristics of the data. Parametric and non-parametric methods were selectively employed based on the distributional characteristics of the variables to compare mean differences. Paired t-tests and Analysis of Variance (ANOVA) were also employed to compare mean differences in health parameters (spleen size, BMI, and hemoglobin level) at different time points, providing further insight into the dataset. This meticulous approach ensured the fidelity of the data analysis process and fortified the reliability of the study's findings. For the survival analysis of the individuals, individuals who got cured of VL were considered events. We employed the Kaplan-Meier approach to estimate the cumulative probability of cure time for the individuals. The Log-rank test was utilized to compare the distinct curves between subgroups. All the tests were two-tailed, and a p-value of <0.05 was considered statistically significant.

## Result

The mean age of the 50 participants in this study was 26.5 years (15.47), among whom 30% (n = 15) were under the age of 18. The study exhibited a balanced gender distribution, with an equal male-to-female ratio among the participants. Furthermore, the geographical distribution indicated a concentration of participants, with nearly three-quarters hailing from Mymensingh, the district with the highest visceral leishmaniasis (VL) burden in Bangladesh. This distribution reflects the prevalence of VL in specific regions (**Table 1)**.

**Table 1:** Sociodemographic distribution of the participants.

Variables	Participants (n)	Percentage (%)
**Age (in years)**	Mean: 26.5	SD: 15.47
**Age group**		
Less than 18 years	15	30%
18 years or more	35	70%
**District**		
Mymensingh	37	74%
Tangail	7	14%
Gazipur	4	8%
Jamalpur	1	2%
Brahmanbaria	1	2%
**Gender**		
Male	25	50%
Female	25	50%
**Occupation**		
Student	17	34%
Housewife	17	34%
Agriculture	7	14%
Businessman	3	6%
Labor	3	6%
Others	3	6%

The clinical features of the participants unveiled a uniform manifestation of the disease. All participants were from VL-endemic regions except one from Brahmanbaria, presenting with symptoms such as fever. Importantly, none had a history of either VL or post-kala-azar dermal leishmaniasis (PKDL). A substantial number of patients (32%) reported a loss of appetite, and a notable proportion (28%) complained of weight loss, whereas more than 90% of the participants fell in the underweight category. Twelve patients experienced abdominal pain, while 20% reported a feeling of abdominal enlargement, and over half of the participants [21] exhibited generalized body weakness. Clinical examination further revealed that all patients had an enlarged spleen, and 18 patients presented with hepatomegaly. Systemic signs of the disease were apparent, with almost 80% of patients displaying pallor and three individuals suffering from jaundice. None of the patients had dehydration, respiratory infections, or lymph node enlargement **(Table 2)**.

**Table 2:** Distribution of clinical features among the patients.

Variables	Participants (n)	Percentage (%)
**Signs & Symptoms**		
Previous H/O VL	0	0%
Previous H/O PKDL	0	0%
Residing in the VL Endemic region	50	100%
Fever	50	100%
Loss of appetite	32	64%
Decreased weight	14	28%
Darkening of skin	04	08%
Nose bleeding	02	04%
Per rectal bleeding	01	02%
Abdominal pain	12	24%
Abdominal fullness	10	20%
Weakness	26	52%
Pallor	39	78%
Jaundice	03	06%
Splenomegaly	50	100%
Hepatomegaly	18	36%
**Body Mass Index (BMI)**		
Underweight (<18.5 kg/m^2^)	46	92%
Normal (≥18.5 kg/m^2^)	04	08%

**Fig 1:**
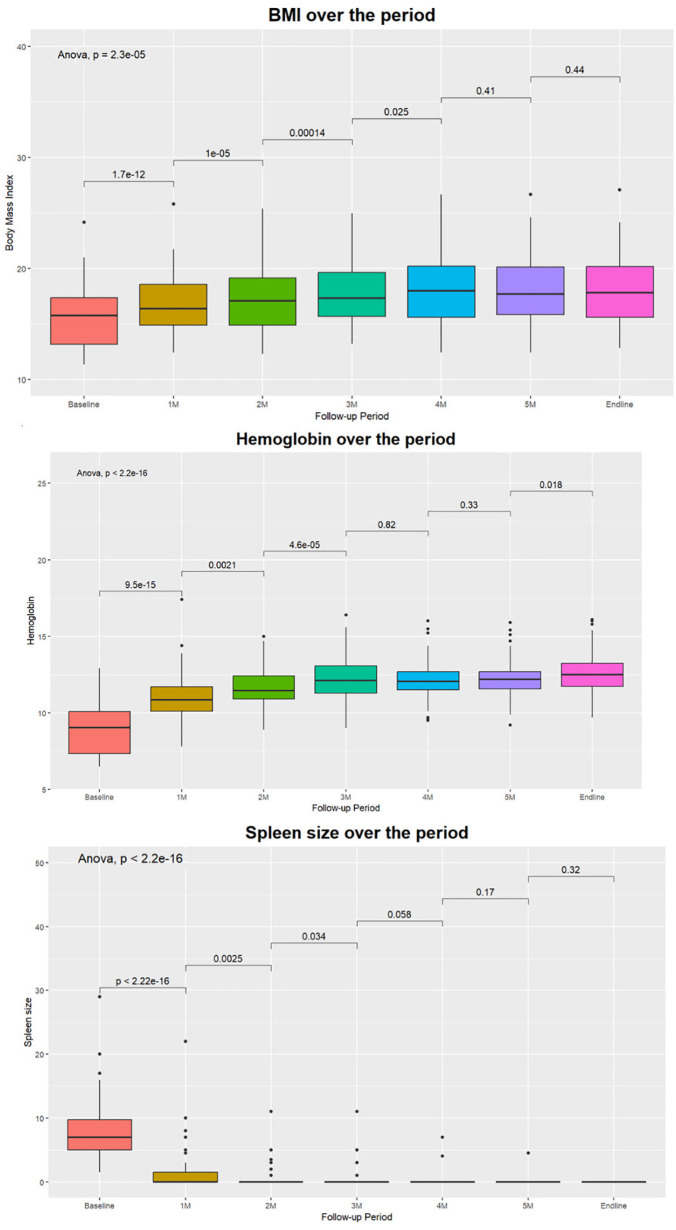
Changes in health parameters over the period (A) BMI, (B) Hemoglobin, (C) Spleen Size.

The study employed several methods to evaluate the changes in several health parameters across different time points within the participant cohort, unveiling a compelling narrative of positive health transformations. The paired t-test results indicate significant improvements in BMI, spleen size, and hemoglobin levels. In the realm of nutrition, there were substantial increases in BMI (Fig 1A) and a notable decrease in the proportion of underweight participants. Hemoglobin (Fig 1B) levels significantly improved in the early months (up to the 3^rd^ month), indicating positive impacts on blood parameters. Equally noteworthy is the pronounced reduction in spleen size over the intervention period, with splenomegaly completely resolved in all the patients by the study's conclusion (Fig 1C). All the enrolled VL patients tested negative in PCR one month after treatment completion and remained negative for six months.

**Fig 2:**
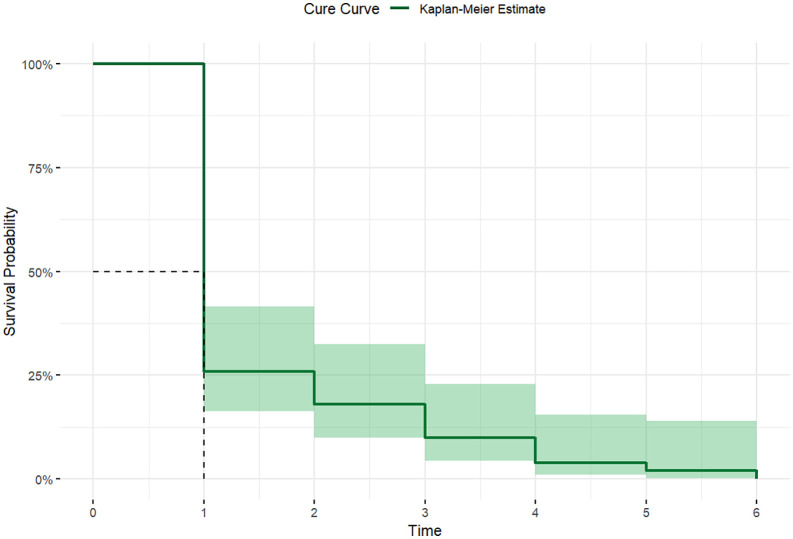
Kaplan-Meier survival estimate for the period of cure from Visceral Leishmaniasis (VL).

The median cure time of the studied population (*n*  =  50) was one month (IQR: 1–2 months), with a cure rate of 100%. Ninety percent (*n*  = 45) of the patients were cured within the 3^rd^ month of treatment completion (Fig 2). However, a significant difference was observed using the Log-rank test (*p*= 0.0002) between individuals with spleen enlargement extending less than or more than 8 cm below the left costal margin (S1 Fig).

## Discussion

This study intended to determine the definitive cure time point for treated VL in the post-elimination phase in Bangladesh. Our findings revealed a 100% cure rate among the study participants, which aligns with previous findings. A single dose of 10 mg/kg L-AmB has shown high efficacy in the Indian subcontinent, with cure rates of 95.7% at six months in one study [22] and 94% at 12 months in another larger study [23]. A previous study showed that LAmB has a 98% cure rate, which is highly acceptable and feasible to dispense in the rural healthcare settings of Bangladesh [24,21]. However, in East Africa, VL is less responsive to LAmB. A single dose of 10 mg/kg resulted in a lower cure rate of 58% at six months [25]. The comparatively lower sensitivity to LAmB reported in East Africa may not only be attributable to differences in *Leishmania* species but also to host-related factors such as more severe clinical presentation, higher rates of malnutrition, HIV co-infection, and greater overall disease burden in that region. None of the participants in this study experienced treatment failure. In a study conducted in India, a single dose of 7.5 mg/kg LAmB resulted in a treatment failure rate of about 2% (4 out of 203 patients) at the initial follow-up [26]. Co-infections, such as HIV or tuberculosis, can significantly increase the risk of treatment failure [27]. In Ethiopia, where VL-HIV co-infection is common, L-AmB monotherapy showed limited effectiveness with an initial parasitological failure rate of 32.8% [27]. As none of our study participants had any co-infections, this might be a reason for zero treatment failure. The uniformity of treatment outcomes in our study reinforces the relevance of assessing the time required to achieve complete clinical and parasitological recovery, which is central to optimizing follow-up schedules in the post-elimination phase.

The mean age of the study participants aligns with previous VL-related studies in Bangladesh. Adults were the predominant age group in this study, contrary to Carvalho et al. [28] and Leite et al. [29]. Leite et al. explained that the highest VL incidence in early childhood is linked to an immature immune system, undernourishment and malnutrition, and a lower notion of hygiene, prevention, and prophylaxis concerning VL [29]. Furthermore, Gontijo and Melo et al. described that 80% of those affected are children under ten years of age in Brazilian VL endemic areas [30], which is also opposite to ours.

One notable finding was that all the patients got parasitological clearance in blood qPCR after one month of treatment completion. Parasitological tests are not recommended in the current national guidelines for cure assessment, which might hinder the identification of treatment failure cases. Quantitative real-time polymerase chain reaction (qPCR) could be a viable candidate for solving this problem. qPCR has higher sensitivity and specificity compared to Leishmania-specific nested PCR (Ln-PCR) [19] and conventional microscopic examination for amastigote in clinical specimens [31]. Adopting qPCR as a test of cure for VL cases during the cure assessment will help in the earlier identification of treatment failure cases, thus contributing to the sustenance of VL elimination efforts.

The 6th month after treatment has always been the time to determine the final cure in most phase 3 clinical trials that the WHO has adopted [32]. However, our current study is the first effort to support the claim and validate the definitive cure time point. On the other hand, this study's findings suggest a thoughtful reconsideration of the existing national guidelines (S1 Table) for cure declaration. Our comprehensive investigation, spanning a 6-month post-treatment period, illuminates a remarkable trend wherein 100% of the patients got cured within the initial three months based on the criteria set by the national guideline (considering substantially reduced spleen size or non-palpable spleen). The cure rate, however, stayed at 90% (45) considering the study's cure criteria (complete regression of spleen). The remaining cases [5] had an above-average spleen size, which took a longer time to regress. Nevertheless, the presence of splenomegaly for an extended period post-VL treatment in patients with regressing spleen is considered a sign of cure by the national kala-azar management guideline [14]. This crucial observation challenges the conventional temporal cure timeframe outlined in the current guidelines, suggesting that the effective duration for cure assessment can be shorter than the current one in practice. A declaration of final cure at 3 months post-treatment (without changing the other timepoints in the follow-up schedule) will allow earlier identification of non-responders as well as add an extra follow-up period, ensuring stronger post-treatment VL surveillance. Changing the final cure time point to three months will also require redefining VL relapse. Currently, VL relapse is defined as the reappearance of symptoms beyond six months (current final cure timepoint) after treatment completion. With the next follow-up scheduled at 12 months (six months after cure declaration), relapse diagnosis and treatment initiation can be delayed if patients do not seek care promptly. By modifying the final cure time point with an additional follow-up at three months, relapse will be defined as a reappearance of symptoms after three months. The implications of this change are pivotal with earlier detection and treatment, which can improve patient outcomes by reducing the duration of untreated disease, potentially leading to better survival rates and reduced healthcare costs. The implications of such swift therapeutic responses have broad consequences for clinical practice and healthcare resource allocation to achieve zero transmission of Kala-azar in the post-elimination phase. As our findings suggest that most VL patients in Bangladesh achieve full recovery within three months, this time point should be considered as the final cure assessment point for all the patients treated for VL. However, continued long-term monitoring in addition to three-month follow-up timepoint remains essential to identify late relapses or PKDL cases.

The evidence of expeditious resolution of the disease observed in all the cases will facilitate the national programme to uphold its commitment to prompt diagnosis and treatment for the VL patients. A revised timeline for final cure assessment followed by more frequent follow-up visits will further ensure early diagnosis of VL relapse and PKDL cases, preventing the prolonged VL transmission in the community. Therefore, with the reduced number of VL cases in the post-elimination phase, this revised timeline for VL case management and follow-up can be pivotal to achieve zero VL transmission by 2030.

## Supporting information

S1 Fig Kaplan-Meier survival estimate for the complete resolution of splenomegaly in Visceral Leishmaniasis (VL) patients grouped by spleen size. (DOCX)

S1 Table Current and proposed VL cure assessment and follow-up schedule. (DOCX)
