# Supplementary material for: Investigating the optimum timeline for final cure assessment of treated Visceral Leishmaniasis patients in Bangladesh
Source: PLOS Glob Public Health. 2026 Feb 26;6(2):e0006002. doi: 10.1371/journal.pgph.0006002 (PMC12944797; doi:10.1371/journal.pgph.0006002)
Supplement: S1 Fig — (DOCX) [file pgph.0006002.s001.docx]

**Supplementary file 1**


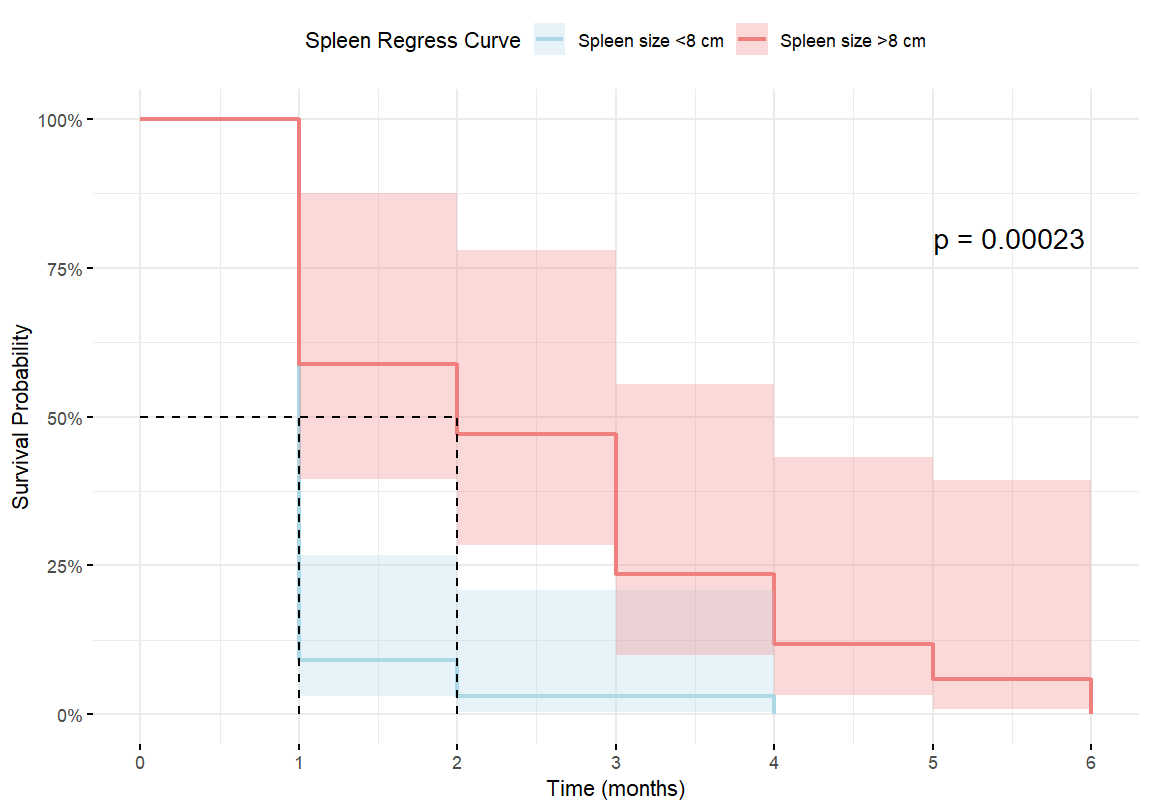


**Fig: Kaplan-Meier survival estimate for the complete resolution of splenomegaly in Visceral Leishmaniasis (VL) patients grouped by spleen size**
