# Supplementary material for: Investigating the optimum timeline for final cure assessment of treated Visceral Leishmaniasis patients in Bangladesh
Source: PLOS Glob Public Health. 2026 Feb 26;6(2):e0006002. doi: 10.1371/journal.pgph.0006002 (PMC12944797; doi:10.1371/journal.pgph.0006002)
Supplement: S1 Table — (DOCX) [file pgph.0006002.s002.docx]

**Supplementary file 1**

|  | **Current schedule** | **Proposed schedule** |
| --- | --- | --- |
| Initial cure assessment | 1 month after treatment | 1 month after treatment |
| Definitive cure assessment | 6 months after treatment | 3 months after treatment |
| Follow-up periods | 12, 24, 36, 48, 60 months after treatment | 6, 12, 24, 36, 48, 60 months after treatment |

**Table: Current and proposed VL cure assessment and follow-up schedule**
